# β-(1→4)-Mannobiose Acts as an Immunostimulatory Molecule in Murine Dendritic Cells by Binding the TLR4/MD-2 Complex

**DOI:** 10.3390/cells10071774

**Published:** 2021-07-14

**Authors:** Ting-Yu Cheng, Yen-Ju Lin, Wataru Saburi, Stefan Vieths, Stephan Scheurer, Stefan Schülke, Masako Toda

**Affiliations:** 1 Laboratory of Food and Biomolecular Science, Graduate School of Agricultural Science, Tohoku University, Sendai 980-8572, Japan; cheng.ting.yu.p2@dc.tohoku.ac.jp; 2 VPr1 Research Group: “Molecular Allergology”, Paul-Ehrlich-Institut, 63225 Langen, Germany; Yen-Ju.Lin@pei.de (Y.-J.L.); Stefan.Vieths@pei.de (S.V.); Stephan.Scheurer@pei.de (S.S.); 3 Research Faculty of Agriculture, Hokkaido University, Sapporo 060-8589, Japan; saburiw@chem.agr.hokudai.ac.jp

**Keywords:** mannobiose, dendritic cells, immunostimulation

## Abstract

Some β-mannans, including those in coffee bean and soy, contain a mannose backbone with β-(1→4) bonds. Such mannooligosaccharides could have immunological functions involving direct interaction with immune cells, in addition to acting as prebiotics. This study aimed at assessing the immunological function of mannooligosaccharides with β-(1→4) bond, and elucidating their mechanism of action using bone marrow-derived murine dendritic cells (BMDCs). When BMDCs were stimulated with the mannooligosaccharides, only β-Man-(1→4)-Man significantly induced production of cytokines that included IL-6, IL-10, TNF-α, and IFN-β, and enhanced CD4^+^ T-cell stimulatory capacity. Use of putative receptor inhibitors revealed the binding of β-Man-(1→4)-Man to TLR4/MD2 complex and involvement with the complement C3a receptor (C3aR) for BMDC activation. Interestingly, β-Man-(1→4)-Man prolonged the production of pro-inflammatory cytokines (IL-6 and TNF-α), but not of the IL-10 anti-inflammatory cytokine during extended culture of BMDCs, associated with high glucose consumption. The results suggest that β-Man-(1→4)-Man is an immunostimulatory molecule, and that the promotion of glycolysis could be involved in the production of pro-inflammatory cytokine in β-Man-(1→4)-Man-stimulated BMDCs. This study could contribute to development of immune-boosting functional foods and a novel vaccine adjuvant.

## 1. Introduction

Mannans are D-mannose-based polysaccharides that comprise the outermost layer of the cell wall in plants, fungi, and yeast [1]. Mannans range from pure mannans containing only mannose to glucomannans and galactomannans comprised of mannosyl residues linked by various glucosidic linkages such as α-(1→2)-, α-(1→3)-, α-(1→6)-, β-(1→2)-, and/or β-(1→4)-links bonds [2,3]. The immunological properties of mannans are diverse, depending on their structures and source. For instance, N-linked and O-linked mannans derived from *Candida albicans* induce anti-fungal protective immunity [4]. Mannans are also significant virulence factors associated with the severity and pathogenesis of *Candida* infections [2,3,4]. In contrast, α-mannan from the cell wall of the yeast *Saccharomyces cerevisiae* induces anti-inflammatory responses by inducing the anti-inflammatory cytokine IL-10 and the co-inhibitory molecule programmed death-ligand 1 in dendritic cells (DCs) [5]. β-Galactomannans from soy also appears to induce such anti-inflammatory properties [6]. These observations indicate that structures of mannosyl residues are an important factor determining the immunological properties of mannan.

Mannans are a valuable source of mannooligosaccharides [3]. Physical, chemical, and mechanical methods that include enzymatic hydrolysis, acid hydrolysis, and ultrasonic degradation of mannans have been developed to prepare mannooligosaccharides in food technology [1,3]. In general, non-digestible oligosaccharides are expected to improve gastrointestinal health by inducing both the growth of beneficial bacteria and the production of short-chain fatty acids (SCFAs) by bacteria [7,8]. In addition, a previous study showed that crude mannooligosaccharides or β-Man-(1→4)-Man (β-(1→4)-mannobiose: Man2) stimulates cytokine production in RAW264.7 macrophages, and exerts anti-inflammatory effects in murine models [9,10]. However, the oligomer size of β-(1→4)-mannooligosaccharides that induces a potent immunological response is unknown.

DCs play a central role in immune responses; they are essential as a bridge between innate and acquired immunity [11,12]. DCs are equipped with a diverse array of specialized receptors, including co-stimulatory molecules and pattern-recognition receptors to specifically recognize “non-self” components [13]. This study assessed the function of several mannooligosaccharides with β-(1→4) bonds on bone marrow-derived murine DCs (BMDCs) and their underlying mechanisms. Among the tested mannooligosaccharides, only Man2 was capable to activate BMDCs. Man2 induced production of cytokines including type I interferon with anti-viral and immune-modulating functions in DCs via Toll like receptor (TLR)-4 and complement C3a receptor (C3aR) engagement. In addition, Man2 enhanced the T-cell stimulatory capacity of DCs. The results suggest that Man2 has potential as a vaccine adjuvant and an immunostimulatory component for functional foods.

## 2. Materials and Methods

### 2.1. Preparation of β-Mannooligosaccharides

β-Man-(1→4)-Man (Man2), β-Man-(1→4)-Man2 (Man3), β-Man-(1→4)-Man3 (Man4), β-Man-(1→4)-Man4 (Man5), β-Man-(1→4)-Glc (Manβ-4Glc), and β-Glc-(1→4)-Man (Glcβ-4Man), were prepared as described previously [14,15]. Preparation of β-Man-(1→4)-GlcNAc (Manβ-4 GlcNAc) is described in the online repository.

### 2.2. Animals

Female C57BL/6J or BALB/c mice were purchased from either Japan SLC, Inc., or Jackson Laboratories (Bar Harbor, ME, USA). OT-II mice were provided by Prof. Ishi Naoto (Tohoku University). The mice were housed under pathogen free conditions. All animal experiments were performed in accordance with either the rules on animal experiments at Tohoku University (2019AgA-026) or the German animal protection law (granting authority: RP Darmstadt).

### 2.3. Cell Culture

Raw264.7 cells were obtained from Riken BioResource Research Center, and maintained in Minimum Essential Media (MEM: Sigma-Aldrich, St. Louis, MO, USA) containing 10% fetal bovine serum (FBS: Sigma-Aldrich, cat. 273012, St. Louis, MO, USA), 0.1 mM non-essential amino acids (NEAA: FUJIFILM Wako Pure Chemical Corporation, Osaka, Japan), 100 units/mL penicillin, and 100 μg/mL streptomycin (Gibco). The cells were passaged using 0.25% trypsin + 0.02% EDTA solution (FUJIFILM Wako Pure Chemical Corporation, Osaka, Japan) to adjust cell density at 3 × 10^5^ cells/mL twice a week. BMDCs were generated by culturing BM cells from mice in RPMI 1640 medium (Sigma-Aldrich, St. Louis, MO, USA) containing 10% FBS, 1 mM sodium pyruvate (Sigma-Aldrich, St. Louis, MO, USA), 10 mM HEPES (Sigma-Aldrich, St. Louis, MO, USA), 0.1 mM 2-mercaptoethanol (Sigma-Aldrich, St. Louis, MO, USA), 100 units/mL penicillin, 100 μg/mL streptomycin, and 20 to 50 ng/mL of rGM-CSF (Peprotech, Rocky Hill, NJ, USA) for 8 days [16]. Approximately 2.0 to 3.0 × 10^7^ of BM cells were collected from one mouse (C57BL/6 or BALB/c mice, female, age of three to four months), and 4.0 to 6.0 × 10^7^ of BMDCs were generated.

### 2.4. Cell Stimulation Assay

Raw264.7 cells or BMDCs (1 × 10^6^ cells/mL) were stimulated with the indicated concentrations of β-mannooligosaccharide or either 5.0 ng/mL or 1.0 μg/mL of lipopolysaccharide from *Salmonella enterica* serotype abortus equi (LPS: Sigma-Aldrich, L5886, St. Louis, MO, USA) in 12-well plates (total medium amount: 1 mL) or 24-well plates (total medium amount: 0.5 mL). To identify receptors on BMDCs binding Man2, the cells were treated for 30 min before the addition of 50 μM Man2 with following inhibitors: 100 nM TAK-242 (Merck Millipore, Burlington, MA, USA), 50 μM C34 (Sigma-Aldrich, St. Louis, MO, USA), 100 μM SB290157 (C3 receptor inhibitor, Sigma-Aldrich, St. Louis, MO, USA), 100 μg/mL of WGP^®^ Soluble (InVivogen, San Diego, CA, USA), or 200 μg/mL of mannan (Sigma-Aldrich, St. Louis, MO, USA). In addition, Cell Counting Kit-8 (CCK-8) assay using WST-8 (2-(2-methoxy-4-nitrophen yl)-3-(4-nitrophenyl)-5-(2,4-disulfophenyl)-2H-tetrazolium, monosodium salt) (Dojindo Laboratories) was performed to assess the influence of receptor inhibitors on viability of BMDCs.

### 2.5. Fluorescence-Activated Cell Sorting (FACS) Analysis

To detect the expression levels of co-stimulatory molecules on the cell surface of RAW-267.4 cells and BMDCs, fluorescein isothiocyanate (FITC)-conjugated rat anti-mouse CD40 (clone 3/23, Biolegend, San Diego, CA, USA), CD80 (clone 16-10A1, Biolegend, San Diego, CA, USA), CD86 (clone GL-1, Biolegend, San Diego, CA, USA), or MHC class II (I-A/I-E) mAbs (clone M5/114.15.2, Biolegend, San Diego, CA, USA) were used. In all stainings, IgG receptors on the cell surface were blocked using either monoclonal antibody (mAb) against CD16/CD32 (clone 2.4G2, Thermo Fisher Scientific, Waltham, MA, USA), or normal murine IgG antibodies (Santa Cruz Biotechnology, Dallas, TX, USA). BMDCs were additionally stained with allophycocyanin (APC)-conjugated anti-mouse CD11c mAb (clone N418, Biolegend, San Diego, CA, USA) to gate the DC population. Fluorescence intensity of stained cells (10,000 cell detection) was measured with flow cytometry using a FACSLyric (BD Bioscience, Franklin Lakes, NJ, USA). Data were analyzed using FlowJo V. 7 (BD Bioscience, Franklin Lakes, NJ, USA).

### 2.6. Cytokine ELISA

Cytokine concentrations in cell culture supernatants were measured by ELISA, as previously described [16]. For more detailed information, see a Supplemental Information File.

### 2.7. SDS-PAGE and Western Blotting

BMDCs (1 × 10^6^ cells/mL: total 1 mL) were stimulated with 1.0 to 50 μM Man2 or 10 μg/mL of LPS for 30 min and lysed in lysis buffer (62.5  mM Tris-HCl (pH 6.8 at 25 °C), 2% *w*/*v* SDS, 10% glycerol, 50  mM DTT, and 0.01% *w*/*v* bromophenol blue) for 10 min on ice. Target proteins in lysates were separated by 15% Tris-glycine SDS-polyacrylamide gels prepared with 30% acrylamide/bis-solution (Roth, Karlsruhe, Germany), and transferred to nitrocellulose membranes (GE Healthcare Life Sciences, Marlborough, MA, USA) in transfer buffer (25 mM Tris base, 0.2 M glycine, 20% methanol) at 350 mA for 2 h. After blocking with 5% non-fat milk (Roth), the resolved proteins were detected by the following antibodies obtained from Cell Signaling Technologies: nuclear factor-kappa B (NF-κB) pathway sampler kit, phospho-mitogen-activated protein kinase (MAPK) family antibody sampler kit, mammalian target of rapamycin (mTOR) substrates antibody sampler kit, and loading control anti-histone H3 antibody. Detection was performed with the provided secondary antibodies using ACE Glow substrate (VWR International GmbH, Darmstadt, Germany). Images were captured with iBright™ CL1500 system (Thermo Fischer Scientific, Waltham, MA, USA) and the intensities of protein bands were quantitated by ImageJ software v1.52a (www.imagej.nih.gov, accessed on 1 August 2020, National Institutes of Health, Bethesda, MD, USA).

### 2.8. Analysis of Metabolic State of BMDCs

The Warburg effect in stimulated BMDC cultures was determined photometrically 72 h post stimulation with the indicated concentrations of 1.0 to 50 μM Man2 and 10 μg/mL of LPS by quantifying the optical density (OD) of culture supernatants at 570  nm by SpectraMAX340PC (Molecular Devices, San Jose, CA, USA) and calculating the Warburg effect as 1/OD570 normalized to unstimulated controls. Glucose concentrations in culture supernatants were determined 72 h post-stimulation using the Glucose (GO) Assay Kit (Sigma-Aldrich, St. Louis, MO, USA). The metabolic rate was derived mathematically from the measured glucose concentrations by calculating the glucose consumption in % in relation to medium without BMDCs (glucose conc. in RPMI1640 = 2  mg/mL).

### 2.9. Assessment of T-Cell Stimulatory Capacity of Man2-Stimulated BMDCs

Splenic CD4^+^ T-cells were isolated from OT-II mice using CD4 (L3T4) MicroBeads (Miltenyi Biotec., Bergisch Gladbach, Germany). To evaluate T-cell activation, CD4^+^ T-cells (8.0 × 10^5^ cells/mL) and BMDCs (1.6 × 10^5^ cells/mL) were seeded in 96-well plates (total medium amount: 0.2 mL), and stimulated with Man2 and LPS-free ovalbumin (OVA: Seikagaku Cooperation, Chiyoda-Ku, Japan). The supernatants were harvested after 24 h for IL-2 determination by ELISA.

### 2.10. Statistical Analysis

Differences between mean values were assessed by Dunnett’s test or two-way ANOVA tests with confidence intervals adjusted for multiple comparisons according to either Bonferroni or Tukey. Statistical analysis was performed with GraphPad Prism v6 to v8 (GraphPad Software, San Diego, CA, USA) and Bell Curve for Excel (Social Survey Research Information Co., Ltd., Shinjuku-ku, Japan). A *p* value of <0.05 was considered significant.

## 3. Results

### 3.1. Man2 Induces Expression of Co-Stimulatory Molecule and Production of Cytokine in RAW264.7 Cells

**Figure 1:**
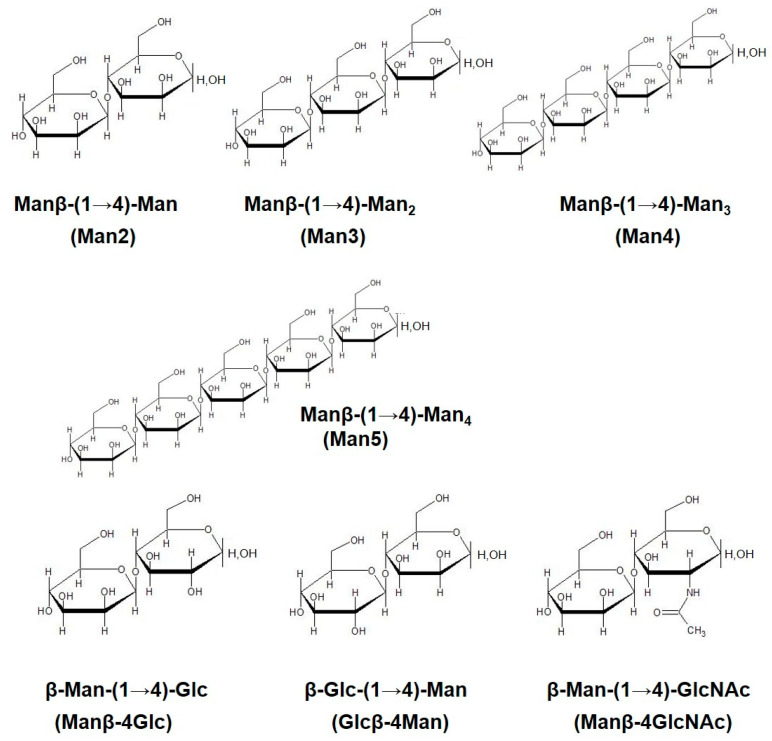
The structure of the tested mannooligosaccharides. Tested samples include β-Man-(1→4)-Man (Man2), β-Man-(1→4)-Man2 (Man3), β-Man-(1→4)-Man3 (Man4), β-Man-(1→4)-Man4 (Man5), β-Man-(1→4)-Glc (Manβ-4Glc), β-Glc-(1→4)-Man (Glcβ-4Man), β-Man-(1→4)-GlcNAc (Manβ-4-GlcNAc).

To assess the effect of mannooligosaccharides with β-(1→4)-linkage on antigen presenting cells, RAW264.7 cells, a murine macrophage cell culture model, were stimulated with the oligosaccharides. Upon stimulation, expression levels of a co-stimulatory molecule (CD40, an activation marker) and cytokine production in the cells were measured. The chemical formulas of the tested mannooligosaccharides are indicated in Figure 1.

**Figure 2:**
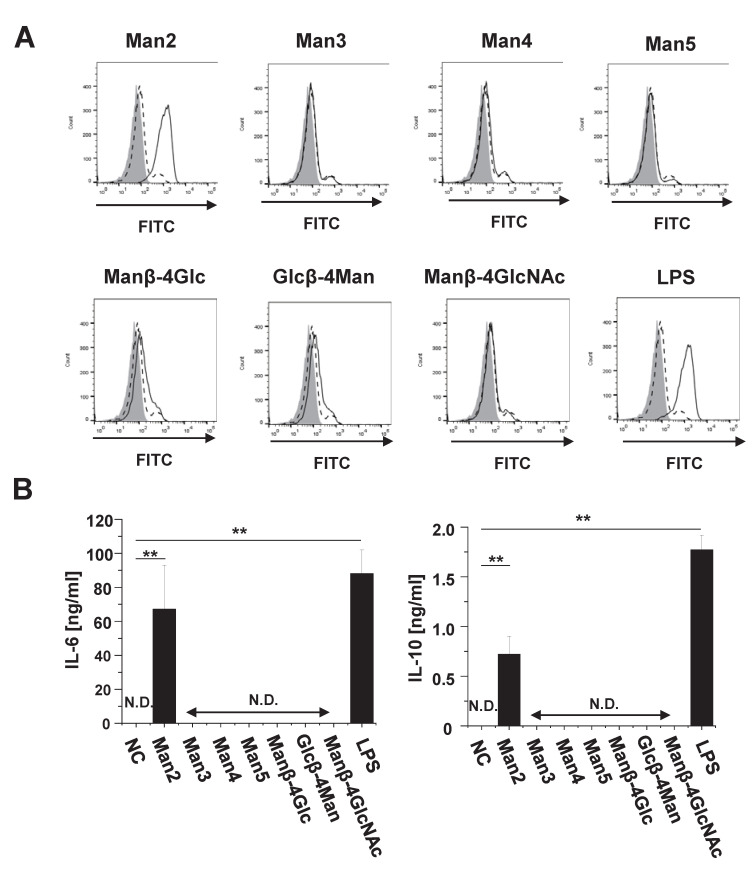
The stimulatory effect of mannooligosaccharides on RAW264.7 cells. (**A**) RAW264.7 cells (1 × 10^6^ cells/mL) were stimulated with either 200 μM of the indicated mannooligosaccharides, 1.0 μg/mL of LPS or cultured only in medium for 24 h. Expression levels of CD40 on the cell surface were analyzed by FACS. Grey area: unstimulated and unstained cells, dashed lines: unstimulated and mAb-stained cells, solid lines: stimulated and mAb-stained cells. (**B**) The concentrations of IL-6 and IL-10 in the cell culture supernatants of RAW264.7 cells (1 × 10^6^ cells/mL) upon stimulation with either 50 μM mannooligosaccharides or 1.0 μg/mL of LPS were determined by ELISA. ** *p* < 0.01 in Dunnett’s test. The data are representative for three independent experiments. All bar graphs show mean ± standard deviation (SD). N.D. (not detectable): <31.25 pg/mL (IL-6 and IL-10).

FACS analysis showed that, in addition to LPS (used as positive control), 50 μM as well as 200 μM Man2 remarkably enhanced CD40 expression on the cell surface (Figure 2A and Figure S1). Both Glcβ-4Man and Manβ-4Glc only marginally induced CD40 expression at a concentration of 200 μM, whereas neither Manβ-4GlcNAc, Man3, Man4, nor Man5 did induce detectable levels of CD40 expression (Figure 2A). Man2, but not other mannooligosaccharides, induced production of both the pro-inflammatory cytokine IL-6 and the anti-inflammatory cytokine IL-10 from RAW264.7 cells (Figure 2B). These results indicate that, among the tested mannooligosaccharides, only Man2 was capable to stimulating RAW264.7 cells.

### 3.2. Man2 Enhances T-Cell Stimulatory Capacity of BMDCs

**Figure 3:**
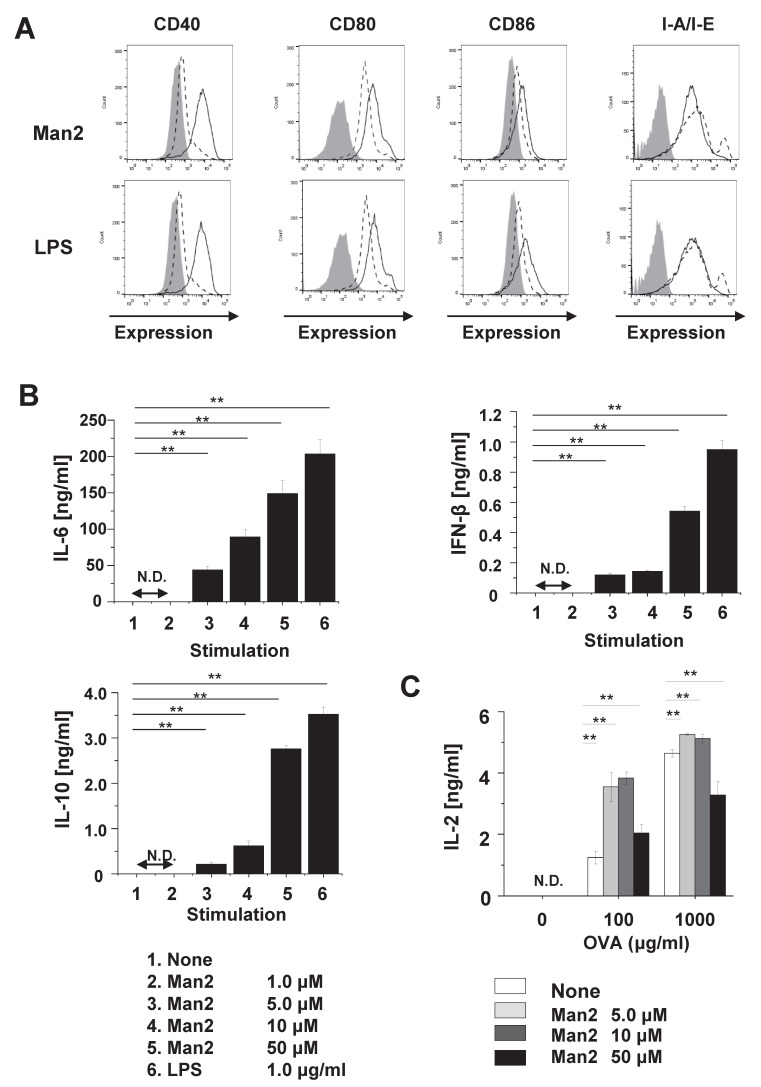
The effect of Man2 on activation and cytokine production of BMDCs. BMDCs (1 × 10^6^ cells/mL) derived from C57BL/6 mice were stimulated with the indicated concentrations of Man2 or 1.0 μg/mL of LPS for 24 h. (**A**) Expression of CD40, CD80, CD86, and MHC class II molecules on the surface of cells upon stimulation with 50 μM Man2 were analyzed by FACS. Grey area: unstimulated and unstained cells, dashed lines: unstimulated and mAb-stained cells, solid lines: stimulated and mAb-stained cells. (**B**) The concentrations of IL-6, IL-10, and IFN-β in the cell culture supernatants were determined by ELISA. (**C**) BMDCs (8.0 × 10^5^ cells/mL) were co-cultured with OT-II cells (1.6 × 10^5^ cells/mL) and stimulated with Man2 and OVA for 24 h. IL-2 concentrations in culture supernatants were measured by ELISA. ** *p* < 0.01 in Dunnett’s test. The data are representative for two independent experiments. All bar graphs show mean ± SD. N.D. (not detectable): <31.25 pg/mL (IL-6 and IL-10), <15.6 pg/mL (IFN-β), <25.0 pg/mL (IL-2).

To assess the effect of Man2 on DC activation, the expression levels of co-stimulatory and MHC class II molecules and cytokine production in BMDCs, primary cultured DCs, were measured 24 h after stimulation with Man2. As well as LPS, Man2 remarkably enhanced the expression of CD40, CD80, and CD86 on the BMDC surface, although this effect was not observed for the expression levels of MHC class II molecules (I-A/I-E) (Figure 3A, Figures S2 and S3). In addition, Man2 induced the production of pro-inflammatory cytokines IL-6 (Figure 3B and Figure S3), TNF-α and IL-1β (Figure S4), anti-inflammatory cytokine IL-10 (Figure 3B and Figure S3), and the anti-viral defense cytokine IFN-β (Figure 3B) in BMDCs. The threshold concentration of Man2 for the induction of detectable levels of CD40 expression and cytokine production in BMDCs, was 1.0 to 5.0 μM (34.43 to 171.2 ng/mL) (Figure 3B, Figures S4 and S5). Other mannooligosaccharides did neither induce the expression of co-stimulatory molecules nor production of cytokines in BMDCs (Figures S2 and S3).

Next, the effect of Man2 on the T-cell stimulatory capacity of BMDCs was assessed in the co-culture system with splenic OT-II cells, i.e., transgenic CD4^+^ T-cells expressing monoclonal OVA-specific T-cell receptors on the C57BL/6 background. Here, stimulation with BMDCs with 5.0 or 10 μM Man2 significantly enhanced IL-2 production by OT-II cells upon stimulation with 100 μg/mL of OVA (Figure 3C). A higher concentration of Man2 (50 μM) and OVA (1000 μg/mL) reduced IL-2 production by OT-II cells. This reduction of IL-2 production is likely due to overstimulation of CD4^+^ T-cells. It is consistent with our previous study showing, that overstimulation of OT-II cells with modified OVA reduced T-cell activation in co-culture with BMDCs [17]. Taken together, these results suggest, that Man2 can enhance the CD4^+^ T-cell stimulatory capacity of BMDCs.

### 3.3. Man2 Activates BMDCs via TLR4 and C3aR Engagement

**Figure 4:**
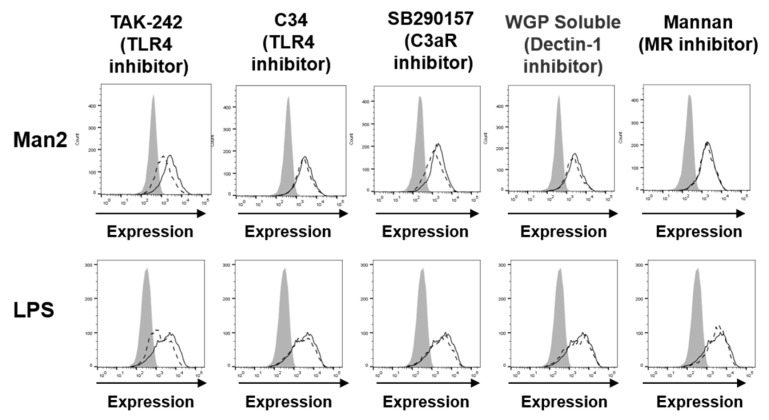
Effect of receptor inhibitors on activation of Man2-treated BMDCs. BMDCs derived from C57BL/6 mice (1 × 10^6^ cells/mL) were treated with either 100 nM TAK-242, 50 μM C34, 100 μM SB290157, 100 μg/mL of WGP^®^ Soluble, or 200 μg/mL of mannan for 30 min, and subsequently stimulated with 50 μM Man2 or 5.0 ng/mL of LPS for 24 h. Expression levels of CD40 on the surface of cells were measured by FACS. Grey lines: unstimulated and unstained cells, dashed lines: inhibitor-and Man2-treated and mAb-stained cells, and black lines: inhibitor-untreated, Man2-treated and mAb-stained cells. The data are representative for two independent experiments.

To elucidate the mechanism of Man2-mediated BMDC activation, we attempted to identify receptor(s) that bound to Man2. BMDCs were pre-treated with different inhibitors of putative receptors, which are known to binds carbohydrates, before Man2 stimulation (Figure 4). TLR-4 inhibition by TAK-242 remarkably suppressed the stimulatory effect of Man2 on CD40, and CD80, CD86 expression in BMDCs (Figure 4 and Figure S6A), although the effect was not observed for I-A/I-E expression. In addition to TAK-242, another TLR4 inhibitor C34, C3aR inhibitor SB290157, Dectin-1 inhibitor WGP^®^ Soluble (whole glucan particle), and the mannose receptor (MR, CD206) inhibitor α-mannan all resulted in statistically significant reduction of CD40 expression in Man2-stimulated BMDCs (Figure S6B), but the changes in the expression were low.

**Figure 5:**
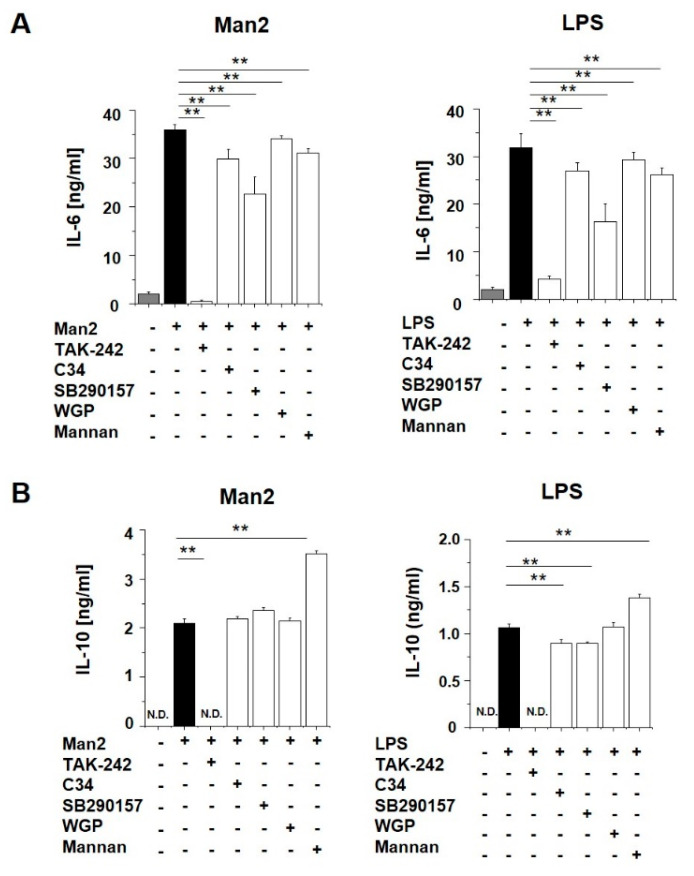
Effect of receptor inhibitors on IL-6 and IL-10 production of Man2-treated BMDCs. BMDCs derived from C57BL/6 mice (1 × 10^6^ cells/mL) were treated with either 10 μM TAK-242, 50 μM C34, 100 μM SB290157, 100 μg/mL of WGP^®^ Soluble, or 200 μg/mL of mannan for 30 min, and subsequently stimulated with 10 μM Man2 or 1.0 μg/mL of LPS for 24 h. The concentrations of (**A**) IL-6 and (**B**) IL-10 in the cell culture supernatants were determined by ELISA. ** *p* < 0.01 in Dunnett’s test. The data are representative for two independent experiments. All bar graphs show mean ± SD. N.D. (not detectable): <31.25 pg/mL (IL-6 and IL-10).

TAK-242 also significantly reduced Man2-induced IL-6, IL-10, TNF-α, IL-1β, and IFN-β production (Figure 5, Figure S7 and S8). C34 and SB290157 moderately reduced Man2-induced IL-6, TNF-α, IL-1β, and IFN-β production (Figure 5A, Figures S7 and S8), but did not affect IL-10 production in Man2-stimulated BMDCs (Figure 5B). The TLR4- and C3aR-inhibitors also reduced cytokine production in LPS-stimulated BMDCs (Figure 5 and Figure S8). Statistical analysis indicated that both WGP^®^ Soluble and α-mannan suppressed IL-6 production in Man2- or LPS-stimulated BMDCs (Figure 5A). However, the reduction level by WGP is very moderate and probably lacks biological significance. The WGP^®^ Soluble did not suppress IL-10 production in the BMDCs (Figure 5B), whereas α-mannan enhanced it (Figure 5B). The viability assay based on WST-8 (2-(2-methoxy-4-nitrophenyl)-3-(4-nitrophenyl)-5-(2,4-disulfophenyl)-2H-tetrazolium, monosodium salt) showed, that the mannooligosaccharides and the inhibitors did not reduce the viability of BMDCs at the tested concentrations (Figure S9). These results suggest, that TLR4 and C3aR are involved in Man2-stimulated BMDC activation.

### 3.4. Man2 Activates MAPKs and NF-κB in BMDCs

**Figure 6:**
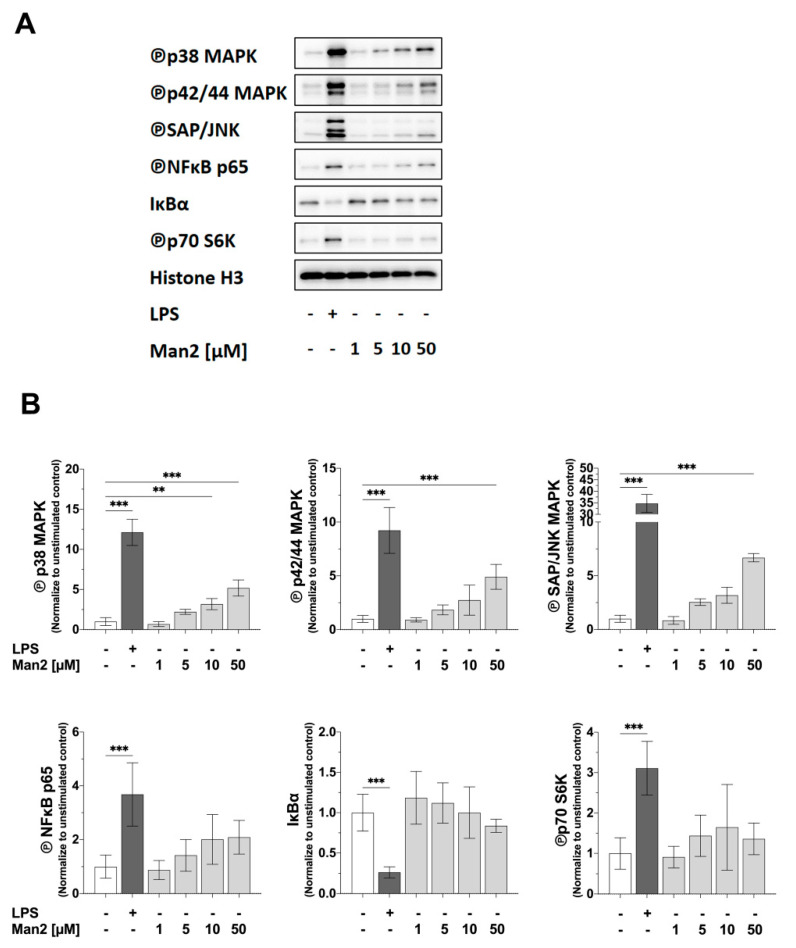
Effect of Man2 on activation of intracellular MAPK-, NF𝜅B-, and mTOR-signaling pathways in BMDCs. BMDCs derived from C57BL/6 mice (1 × 10^6^ cells/mL) were stimulated with either 1.0 to 50 μM Man2 or 10 μg/mL of LPS. (**A**) After 30 min of stimulation, 1 × 10^6^ cells were lysed and target proteins in lysates were detected by Western blotting. (**B**) The intensities of protein bands were quantitated by Image J software. Data are mean values  ±  SD of four independent experiments. ** *p* < 0.01, *** *p* < 0.001 in 2-way ANOVA tests.

To gain insight into the mechanism of Man2-driven activation of BMDCs, we stimulated the cells with either Man2 or LPS and analyzed the signaling pathways (Figure 6). LPS induced the phosphorylation of p38 kinase, p42/44 kinases, stress-activated protein kinase/c-Jun N-terminal kinase (SAP/JNK), NF-κB/p65, and mTOR-related p70 ribosomal protein S6 kinase (p70S6K), and reduced expression of NF-κB inhibitor α (IkBα) (Figure 6). Man2 also induced dose-dependently higher phosphorylation of all tested MAPKs (p38, p42/44 kinases, and SAP/JNK). The levels of phosphorylation of the tested kinases (except NFkB/p65 and p70S6K) in BMDCs stimulated at 50 μM Man2 were significantly higher than those in non-stimulated cells. Man2 induced dose-dependently, but not significantly higher phosphorylation of NF-kB/p65, and reduced IkBα expression, while phosphorylation of p70S6K was induced only marginally (Figure 6). These results suggest that Man2 mainly activates the kinase- and NF-kB-related signaling cascades, but not the mTOR/p70S6K cascade, in contrast to this LPS activates all these cascades.

### 3.5. Man2 Activates Glucose Metabolism in BMDCs

**Figure 7:**
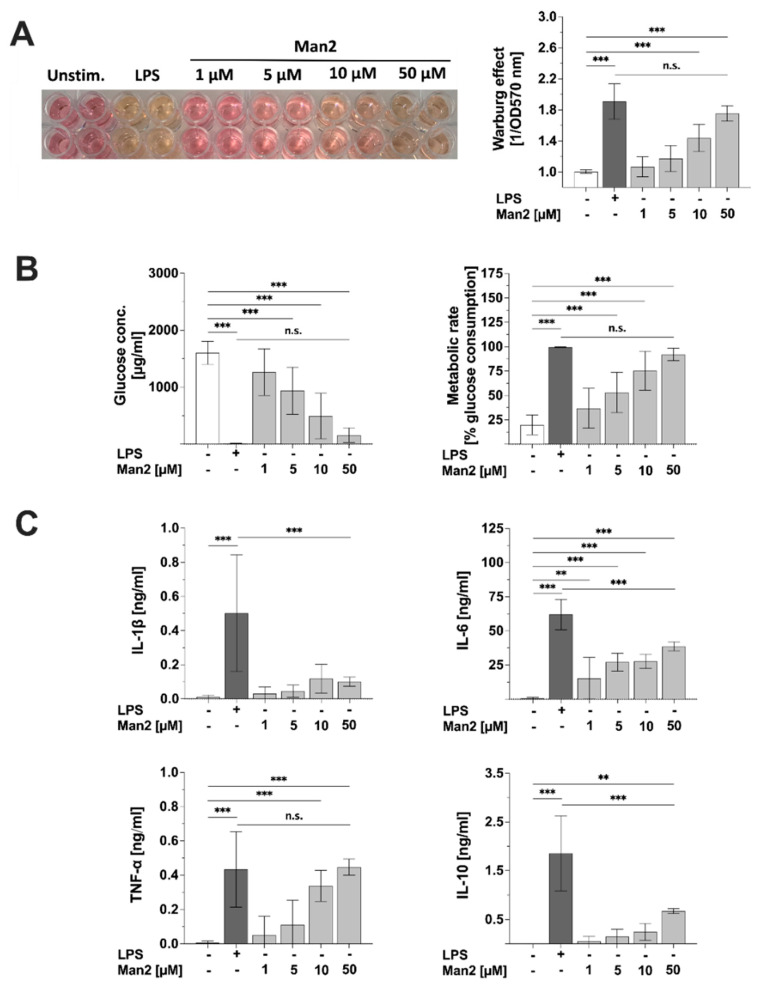
Effect of Man2 on glucose metabolism and cytokine secretion from BMDCs. BMDCs derived from C57BL/6 mice (1 × 10^6^ cells/mL) were stimulated with either 1.0 to 50 μM Man2 or 10 μg/mL of LPS. (**A**) After 72 h of stimulation, the induced Warburg effect was quantified both optically and photometrically. In addition, (**B**) glucose consumption and metabolic rate and from culture medium were determined, whereas (**C**) cytokine concentrations in culture supernatants were determined by ELISA. Data are mean values  ±  SD of four independent experiments. ** *p* < 0.01, *** *p* < 0.001 in Tukey’s HSD multiple comparison test. n.s. = not significant.

LPS influences the glucose metabolism in BMDCs [18,19]. To analyze the impact of Man2 on glucose metabolism, BMDCs were stimulated with either Man2 or LPS for 72  h and induced Warburg effect, cell metabolic state, and cytokine secretion were analyzed. Interestingly, 50 μM Man2 induced a strong Warburg effect in BMDCs, as observed in LPS-stimulated cells (Figure 7A). Man2 also increased glucose consumption and metabolic rate (Figure 7B). The concentrations of the IL-6 and TNF-α pro-inflammatory cytokines in the culture supernatant of Man2-stimulated cells were comparable to those in LPS-stimulated cells, although Man2-induced levels of IL-1ß were found to not be significantly different from unstimulated cells (Figure 7C). In contrast, the concentration of the IL-10 anti-inflammatory cytokine in the cell culture supernatant of Man2-stimulated cells was significantly lower than that in LPS-stimulated cells (Figure 7C). These results suggest that Man2 prolongs the secretion of pro-inflammatory cytokines, associated with increased glucose metabolism.

## 4. Discussion

In this study, we demonstrated that Man2, but not other tested mannooligosaccharides with the β-1,4-linkage, activates murine BMDCs via both TLR4- and C3aR-engagement and enhances the T-cell stimulatory capacity of the cells. The enhanced IL-2 production by OT-2 cells in a co-culture of Man2-stimulated BMDCs would be associated with up-regulation of co-stimulatory molecules, CD40, CD80, and CD86 on the cell surface of the stimulated DCs. Compared to that for short time frames (24 h), culture of Man2-stimulated BMDCs for extended timeframes (72 h) appeared to retain the secretion of pro-inflammatory cytokines, but reduced the secretion of anti-inflammatory cytokine remarkably. These results suggest that Man2 could be used as an immunostimulatory molecule to activate DCs and enhance subsequent antigen-specific CD4^+^ T-cell activation.

Our previous studies showed that MAPK-mediated activation of both NFκB- and mTOR-signaling likely is a key pathway for IL-10 secretion in TLR5-engaged BMDCs with a flagellin:allergen fusion protein [20,21]. However, in the present study, mechanistically, Man2 activated MAPKs (p38, p42/44, SAP/JNK) and NF-κB at certain levels, but only marginally activated p70S6K in BMDCs. Poncini et al. showed that high levels of IL-10 production are associated with p42/44 and NF-κB signaling in TLR4 engaged DCs with heat-killed trypomastigotes [22]. The results suggest, that Man2 could induce initial IL-10 production mainly via MAPK and NF-κB, but not the mTOR pathway.

In the present study, BMDCs secreted IL-10 during the first 24 h of culture upon stimulation with Man2 with secretion apparently being reduced thereafter. The decreased activation of p70S6K might be linked to the reduced IL-10 secretion in Man2-treated BMDCs. However, in contrast to IL-10 secretion, secretions of IL-6 and TNF-α were detectable after 72 h culture of Man2-treated BMDCs, similar to the observations in LPS-stimulated cells. Several studies have reported TLR-ligands to induce IL-6 and TNF-α production by activating MAPKs and NF-κB in BMDCs [16,21,23]. In addition, recent studies described that a shift from oxidative phosphorylation to anaerobic glycolysis in the metabolism of macrophages and DCs is important for pro-inflammatory cytokine production and effector function of the respective cells [18,19]. Interestingly, a potent glucose metabolism characterized by induction of the Warburg effect and increased glucose consumption from the culture medium was also detected in either LPS- or Man2-stimulated BMDCs. These results suggest that, in addition to the activation of MAPKs and NF-κB, pronounced glucose metabolism is associated with pro-longed IL-6 and TNF-α secretion in Man2-stimulated BMDCs.

To assess the involvement of TLR4 in Man2-mediated cell activation, we pre-treated BMDCs with the two TLR-4 inhibitors TAK-242 [24], and C34 [25]. TAK-242 remarkably reduced Man2-mediated CD40 expression and cytokine production in BMDCs. This is consistent with the previous observation that TAK-242 inhibited Man2-mediated TNF-α production in RAW264.7 cells [10]. TAK-242 is a small-molecule compound that selectively inhibits TLR4 signaling by binding to its intracellular domain [24]. C34 inhibits TLR4 signaling in a different manner by binding to a hydrophobic pocket of myeloid differentiation protein-2 (MD-2), a co-receptor of TLR4 expressed on the cell surface [25]. Although the inhibitory effects of C34 on Man2-mediated BMDC activation were moderate, this MD-2 inhibitor reduced Man2-, or LPS-induced cytokine production in BMDCs. LPS binds the MD-2/TLR4 complex via lipid A molecule, thereby triggering the activation of downstream signaling pathways of TLR4 [26,27]. Several studies have shown that various types of small molecules, such as palmitic acid and neoseptins (chemically synthesized peptidomimetics) primarily bind to the TLR4-MD2 complex and triggers TLR4-mediated signaling in both DCs and macrophages [28,29]. Taken together, the findings indicate, that it is likely that Man2 binds to the TLR4/MD-2 complex, and triggers TLR4-mediated signaling and activation of BMDCs.

It is interesting that only Man 2, but not other tested mannooligosaccharides, is capable of inducing BMDC activation. Compared to galactose and glucose, the hydrophobicity of mannose is higher [30]. In addition, the beta-face of D-mannose is basically hydrophobic, whereas the alpha-face is hydrophilic because OH groups of this hexamer orient in this alpha side due to axial configuration of the C2-hydroxyl group (2-OH) [31]. As described above, some small molecules bind to a hydrophobic pocket of MD-2, and thereby trigger or antagonize activation of TLR4-mediated signals in DCs and macrophages [28,29]. Man2 might trigger BMDC activation by binding the hydrophobic pocket of MD-2 with its hydrophobic beta-face. Other mannooligosaccharides might not be able to bind the pocket of MD-2 due to steric hindrance.

Compared to TAK-242, the inhibitory effect of C34 on Man2-stimulated BMDC activation is low. C34 suppressed IL-6, but not IL-10 production in the cells. In silico analyses showed, that C34 is a hydrophobic compound, and binds mainly to hydrophobic amino acid side chains in the pocket of MD-2, including F121, F119, L61, I117, Y102, I94, V93, F76, V135, and I78, with the exception of E92 [25]. Man2 has hydrophilic alpha-face due to the presence of several OH groups, although its beta-face is hydrophobic. Therefore, the binding sites of C34 and Man2 could be different. C34 may cover only some of the space of Man2-binding sites in MD-2 that is otherwise occupied by Man2, and not inhibit the effect of Man2 effectively.

In addition to TLR4- and MD2-inhibitors, the C3aR inhibitor SB 290157 [32] also reduced Man2-mediated IL-6 production, but not IL-10 production, suggesting that Man2-mediated C3aR engagement is mainly involved in the induction of IL-6 in BMDCs. C3aR is expressed on leukocytes of the myeloid lineages, such as DCs, macrophages, monocytes, neutrophils, basophils, and mast cells. C3aR is a seven-transmembrane G protein-coupled receptor with a large second extracellular loop [33], which plays an important role in the interaction with its ligands including C3a, LPS, and probably Man2. Several studies have shown that C3aR engagement prevents IL-10 production in monocytes, macrophages, and T cells, and induces pro-inflammatory responses dominantly [34,35]. Engagement of the C3a receptor by Man2 might be at least in part involved in the prolonged secretion of IL-6 and the reduction of IL-10 during prolonged culture time of BMDCs.

α-Mannan significantly enhanced Man2-stimulated IL-10 production, while reducing IL-6 production moderately in BMDCs. We intended to use α-mannan as an inhibitor of the mannose receptor initially. However, Sirvent et al. showed that α-mannan induces IL-10 production predominantly in human DCs via DC-SIGN (Dendritic Cell-specific ICAM-3 Grabbing Non-integrin: CD209) engagement [6]. IL-10 inhibits the function of APCs, including production of pro-inflammatory cytokines [36]. It is likely that α-mannan enhanced IL-10 production and thereby suppressed that IL-6 production in Man2-stimulated BMDCs.

In conclusion, an exclusive stimulatory effect of Man2 on DC activation was demonstrated in an *in vitro* culture system. This is the first study to show, that Man2-drives DC activation occurring via binding of TLR4 and C3aR and subsequent intracellular signaling events. Our results suggest, that Man2 is an immunostimulatory molecule inducing production of pro-inflammatory cytokines and type I IFN in DCs. Man2 also enhances T-cell stimulatory capacity of BMDCs. The results suggest, that Man2 has potential as a vaccine adjuvant and an immunostimulatory component for functional foods. Further studies, e.g., in vivo immunization, or feeding study would be necessary to establish the application of Man2 for health benefits.

## Data Availability

The data that support the findings of this study are available from the corresponding authors, upon reasonable request.

## Supplementary Materials

The following are available online at https://www.mdpi.com/article/10.3390/cells10071774/s1, Materials and Methods, Figure S1. Stimulatory effect of mannooligosaccharides on RAW264.7 cells. Figure S2. Stimulatory effect of mannooligosaccharides on expressions of co-stimulatory and MHC class II molecules in BMDCs. Figure S3. Dose dependent effect of Man2 on CD40 expression in BMDCs. Figure S4. Stimulatory effect of mannooligosaccharides on cytokine production in BMDCs. Figure S5. Stimulatory effect of Man2 on TNF-α and IL-1β production in BMDCs. Figure S6. Effect of receptor inhibitors on expressions of co-stimulatory molecules in Man2-treated BMDCs. Figure S7. Effect of receptor inhibitors on expressions of IFN-β production in Man2-treated BMDCs. Figure S8. Effect of receptor inhibitors on expressions of TNF-α and IL-1β production in Man2-treated BMDCs. Figure S9. The influence of mannooligosaccharides and receptor inhibitors on viability of BMDCs.

## Abbreviations

APCs	Antigen presenting cells
BMDCs	Bone marrow derived murine dendritic cells
C3aR	Complement 3a receptor
FITC	Fluorescein isothiocyanate
mAb	Monoclonal antibody
TLR4	Toll-like receptor 4
Man2	β-Man-(1→4)-Man
Man3	β-Man-(1→4)-Man2
Man4	β-Man-(1→4)-Man3
Man5	β-Man-(1→4)-Man4
Manβ-4Glc	β-Man-(1→4)-Glc
Glcβ-4Man	β-Glc-(1→4)-Man
Manβ-4 GlcNAc	β-Man-(1→4)-GlcNAc
MD-2	myeloid differentiation protein-2
