# Supplementary material for: β-(1→4)-Mannobiose Acts as an Immunostimulatory Molecule in Murine Dendritic Cells by Binding the TLR4/MD-2 Complex
Source: Cells. 2021 Jul 14;10(7):1774. doi: 10.3390/cells10071774 (PMC8305851; doi:10.3390/cells10071774)
Supplement: Supplementary file 1 [file cells-10-01774-s001.zip › cells-1255345-supplementary.pdf]

Supporting information

β-(1→4)- Mannobiose acts as an immunostimulatory molecule in murine dendritic cells by binding the TLR4/MD-2 complex

Cheng Ting-Yu [a], Yen-Ju Lin [b], Wataru Saburi [c], Stefan Vieths [b], Stephan Scheurer [b], Stefan Schülke [b#], Masako Toda [a#]

[a] Laboratory of Food and Biomolecular Science, Graduate School of Agricultural Science, Tohoku University, Sendai, Japan.

[b] VPr1 Research Group: "Molecular Allergology", Paul-Ehrlich-Institut, Langen, Germany.

[c] Research Faculty of Agriculture, Hokkaido University, Sapporo, Japan.

[d] Laboratory of Immunology, Graduate School of Medical school, Tohoku University, Sendai, Japan.

# Equal contribution

Corresponding author

Dr. Masako Toda

Laboratory of Food and Biomolecular Science,

Graduate School of Agricultural Science, Tohoku University, Sendai, Japan.

E-mail: masako.toda.a7@tohoku.ac.jp

Dr. Stefan Schülke

VPr1 Research Group: "Molecular Allergology",

Paul-Ehrlich-Institut, Langen, Germany

E-mail: stefan.schuelke@pei.de

Materials and Methods

Preparation of Manβ-4GlcNAc

Manβ-4GlcNAc was prepared from α-mannose 1-phosphate (α-Man1P) and N-acetyl-D-glucosamine through the reverse phosphorolysis catalyzed by 1,4-β-mannosyl-N-acetylglucosamine phosphorylase (MNP, EC 2.4.1.320). Recombinant MNP from Bacteroides fragilis NCTC 9343 (BfMNP; GenBank number, CAH07033.1) was prepared as described below. The BfMNP gene was amplified from the genomic DNA of B. fragilis NCTC 9343 by PCR using Primestar HS DNA polymerase (Takara Bio, Kusatsu, Japan) and primers, 5´-AATATTAATTTAATCGAATC-3´ (sense) and 5´-CCCTTTCTGTTTTATTCGAT-3´ (antisense). Amplified DNA fragment was used as the template for the second PCR, in which primers, 5´-TTAACTTTAAGAAGGAGATATACATATGGAAGAAATTAAAATTGC-3´ (sense) and 5´-GATCTCAGTGGTGGTGGTGGTGGTGTCAGATGATACTTGTACGTT-3´ (antisense), were used. The PCR product and linear pET-23a, prepared by PCR using primers, 5´-CACCACCACCACCACCACTGAGATC-3´ (sense) and 5´-ATGTATATCTCCTTCTTAAAGTTAA-3´ (antisense), were connected with In-Fusion HD Cloning Kit (Takara Bio). The expression plasmid was propagated in Escherichia coli DH5α. The DNA sequence of the inserted DNA including its flanking regions was determined with an Applied Biosystems 3130 Genetic Analyzer (Foster City, CA, USA).

Recombinant BfMNP was produced in E. coli BL21 (DE3) transformant, harboring the expression plasmid. Bacterial cells were incubated in 2.0 L of LB medium containing 100 μg/mL ampicillin with vigorous shaking at 37°C until OD600 reached 0.6. Production of the recombinant protein was induced by the addition of 0.1 M isopropyl β-thiogalactoside (IPTG) at a final concentration of 0.1 mM, and the induction culture was carried out at

18°C for 24 h. Bacterial cells, harvested by centrifugation at 6,000 ×g at 4°C for 10 min, were disrupted by sonication in 100 mL of 10 mM 2-morpholinoethanesulfonic acid (MES)-NaOH buffer (pH 6.5). Cell-free extract after removal of cell debris by centrifugation at 6,000 ×g at 4°C for 10 min was subjected to anion exchange column chromatography using DEAE Sepharose Fast Flow (2.8 cm I.D. × 20 cm; GE Healthcare, Uppsala, Sweden). After eluting non-adsorbed protein with 10 mM MES-NaOH buffer (pH 6.5), adsorbed protein was eluted by a linear gradient of NaCl from 0 to 0.4 M (total elution volume, 600 mL). The active fraction obtained was further separated by hydrophobic column chromatography using Toyopearl Butyl 650-M (2.8 cm I.D. × 10 cm; Tosoh, Tokyo, Japan). Non-adsorbed protein was eluted with 10 mM MES-NaOH buffer (pH 6.5) containing 25% saturation ammonium sulfate, and adsorbed protein was eluted by a descending linear gradient of ammonium sulfate (25–0% saturation; total elution volume, 600 mL). The fractions containing highly purified BfMNP, judged by SDS-PAGE, were pooled and dialyzed against 10 mM MES-NaOH buffer (pH 6.5).

A reaction mixture (200 mL), containing 7.94 μg/mL BfMNP, 50 mM Man1P, 100 mM N-acetyl-D-glucosamine (Nacalai Tesque, Kyoto, Japan), and 50 mM MES-NaOH buffer (pH 6.5), was incubated at 37°C for 24 h. Man1P bis(cyclohexylammonium) salt, prepared by the publication of Liu et al. [Carbohydrate Research, 401, 1-4 (2015)], was kindly gifted by Dr. Motomitsu Kitaoka (Niigata University, Niigata, Japan). Sample, passed through a membrane filter Amicon Ultra 30,000 nominal molecular weight limit (Merck, Darmstadt, Germany), was concentrated to 20 mL under reduced pressure. The reaction product was purified by gel filtration column chromatography under following conditions: column, Toyopearl HW-40S (5.0 cm I.D. × 95 cm, Tosoh); elution, water; fraction volume, 5 mL. The fractions, containing highly purified Manβ-4GlcNAc, were

pooled and lyophilized (629 mg of Manβ-4GlcNAc was yielded). Chemical structure of Manβ-4GlcNAc was verified by ESI-MS and NMR. NMR spectra were recorded in D2O at 27°C using an AMX500 (500 MHz; Bruker, Billerica, MA, USA). A series of two-dimensional homo- and heteronuclear correlated spectra [correlated spectroscopy, heteronuclear single quantum correlation (HSQC), non-decoupling HSQC, HSQC total correlation spectroscopy, and heteronuclear multiple bond correlation correlated spectroscopy (HMBC)] were acquired.

Cytokine ELISA

The concentrations of cytokines in cell culture supernatants of RAW264.7 cells and BMDCs were measured by ELISA using antibodies and ELISA kits listed below. Following incubation with the respective detection antibodies, ELISA plates were incubated with streptavidin horseradish peroxidase for 30 minutes at room temperature. Detection was performed with 100 μL 3,3′ ,5,5′ -tetramethylbenzidine (Carl Roth Chemikalien, or BioLegend) and incubation at room temperature. The reaction was stopped with 1 M sulfuric acid (Carl Roth Laborbedarf, or FUJIFILM Wako Pure Chemical Corporation). Optical density was measured at 450 nm by SpectraMAX340PC (Molecular Devices), or iMark microplate absorbance reader (Bio-Rad).

<u>List of antibodies and ELISA kits</u>

Purified anti-mouse IL-1β mAb (1:500 dilution: eBioscience, cat.14-7012-85)

Biotin-conjugated anti-mouse IL-1β polyclonal antibody (1:500 dilution: eBioscience, cat.13-7112-81)

Purified anti-mouse IL-6 mAb (1:1000 dilution: eBioscience, cat.14-7061-85)

Biotin-conjugated IL-6 mAb (1:1000 dilution: eBioscience, cat.13-7062-85)

Purified anti-mouse TNF-α (1:500 dilution: eBioscience, cat.14-7325-85)

Biotin-conjugated TNF-α mAb (1:250 dilution: eBioscience, cat. 13-7326-85)

Streptavidin horseradish peroxidase (1:2000 dilution: eBioscience, cat. 554066)

ELISA MAX™ Standard Set Mouse IL-1β (BioLegend, cat. 432601)

ELISA MAX™ Standard Set Mouse IL-6 (BioLegend, cat.431301)

ELISA MAX™ Standard Set Mouse IL-10 (BioLegend, cat. 431411)

Mouse IL-10 ELISA Development Kit (PeproTech, cat.900-T53)

ELISA MAX™ Standard Set Mouse TNF-α (BioLegend, cat. 430901)

Mouse IFN-beta DuoSet ELISA (R&D systems: cat. DY8234-05)

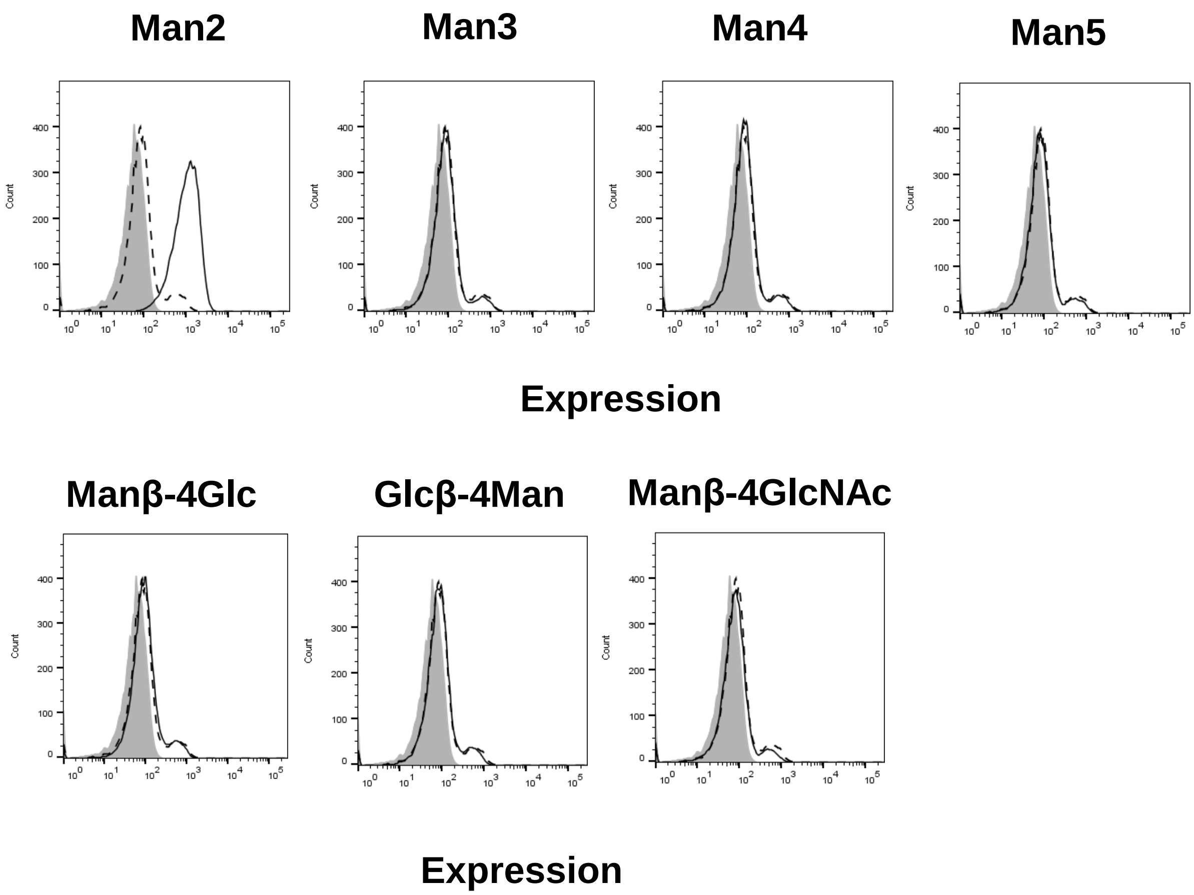

Fig. S1. Stimulatory effect of mannooligosaccharides on CD40 expression in RAW264.7 cells. RAW264.7 cells ($1x10^6$ cells/mL) were stimulated with 50 μM of the indicated mannooligosaccharides or 1.0 μg/mL of LPS. Expression levels of CD40 on the cell surface were analyzed by FACS. Grey area: unstimulated and unstained cells, dashed lines: unstimulated and mAb-stained cells, solid lines: stimulated and mAb-stained cells. The data are representative for two independent experiments.

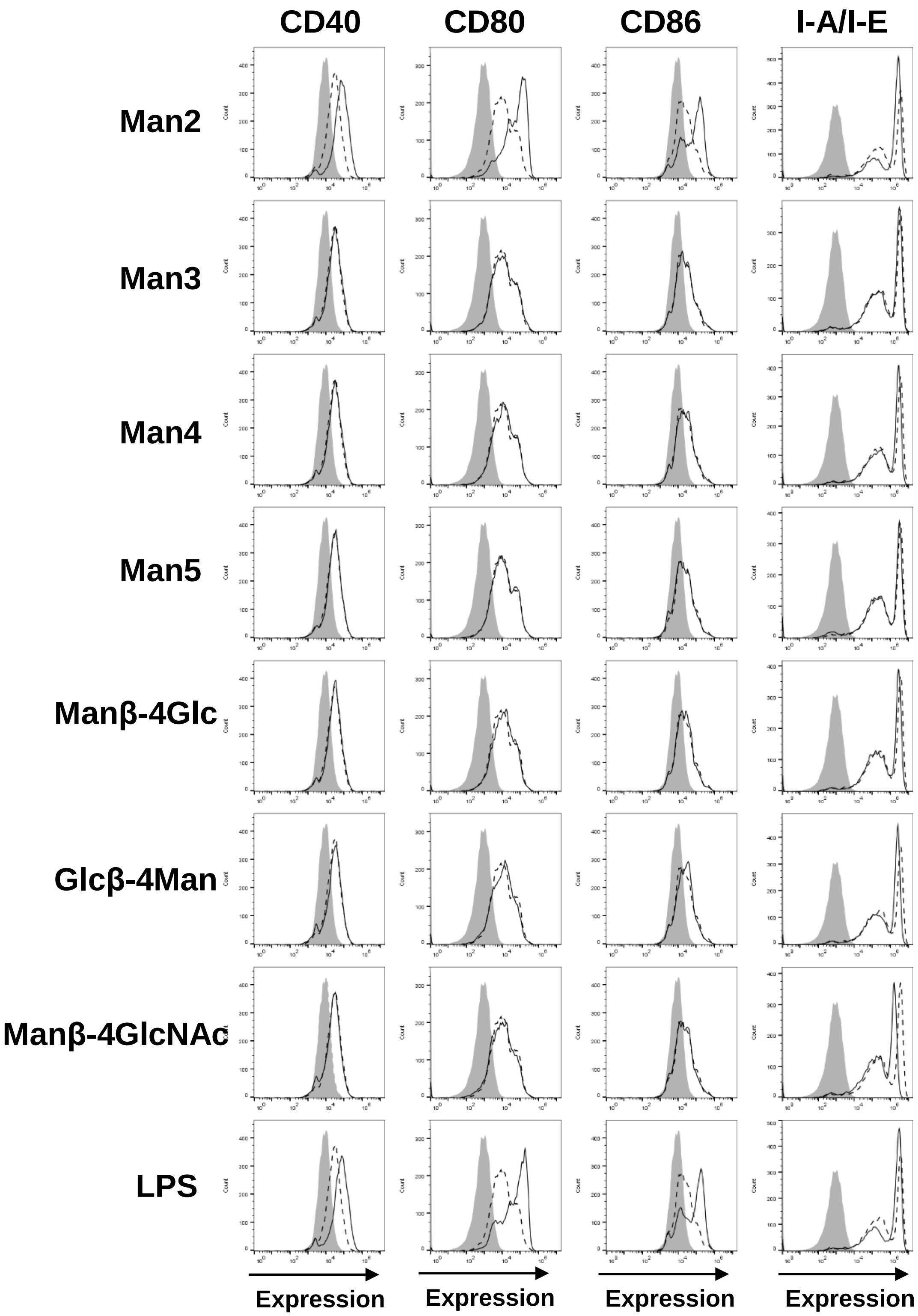

Fig. S2. Stimulatory effect of mannooligosaccharides on expressions of co-stimulatory and MHC class II molecules in BMDCs. BMDCs from BALB/c mice ($1x10^6$ cells/mL) were stimulated with 50 μM of the indicated mannooligosaccharides or 1.0 μg/mL of LPS. Expression levels of CD40, CD80, CD86 and I-A/I-E on the cell surface were analyzed by FACS. Grey area: unstimulated and unstained cells, dashed lines: unstimulated and mAb-stained cells, solid lines: stimulated and mAb-stained cells. The data are representative for two independent experiments.

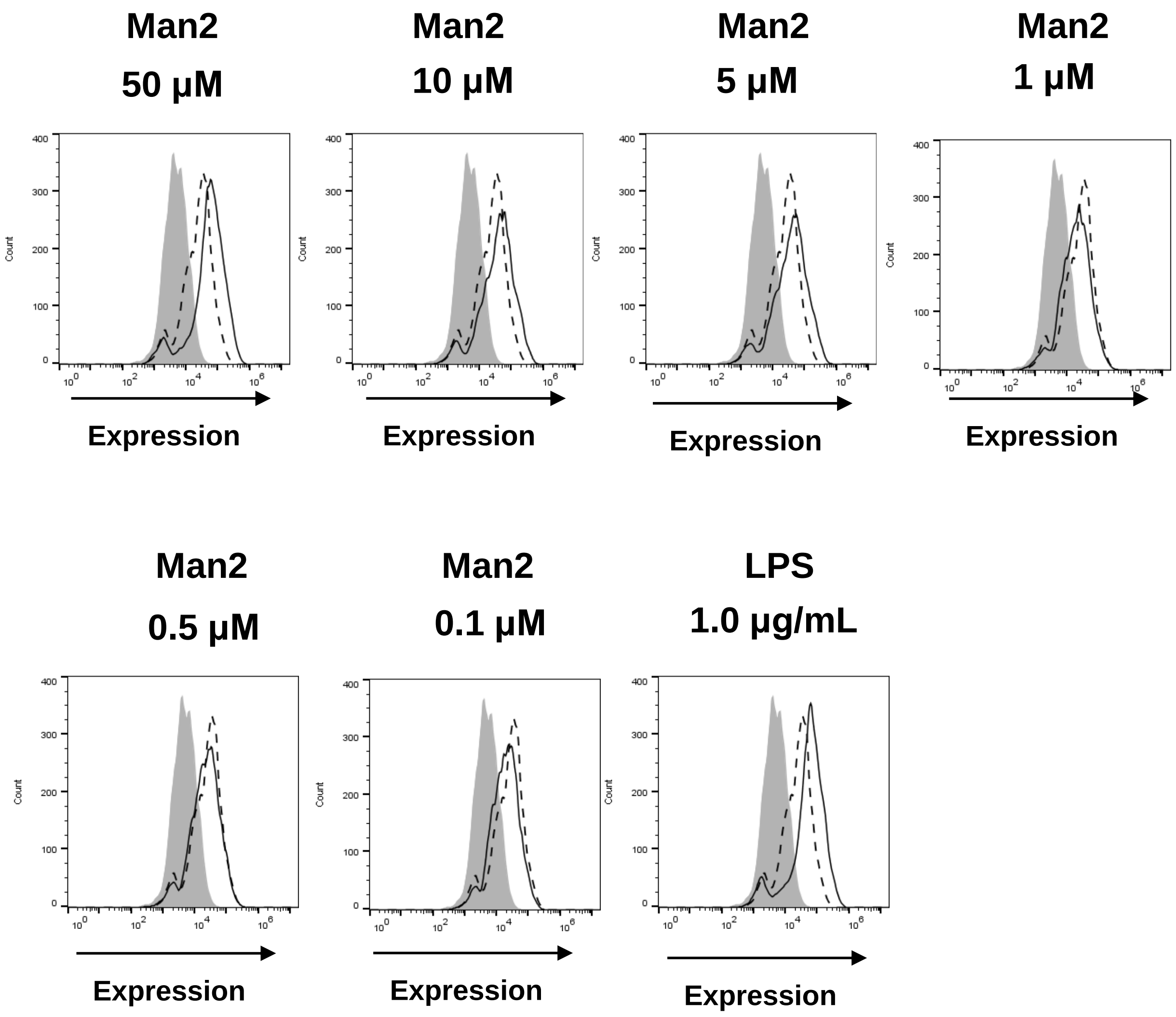

Fig. S3. Dose dependent effect of Man2 on CD40 expression in BMDCs. BMDCs derived from BALB/c mice ($1x10^6$ cells/mL) were stimulated with different concentrations of Man2, or 1.0 µg/mL of LPS. Expression levels of CD40 on the cell surface were analyzed by FACS. Grey area: unstimulated and unstained cells, dashed lines: unstimulated and mAb-stained cells, solid lines: stimulated and mAb-stained cells. The data are representative for two independent experiments.

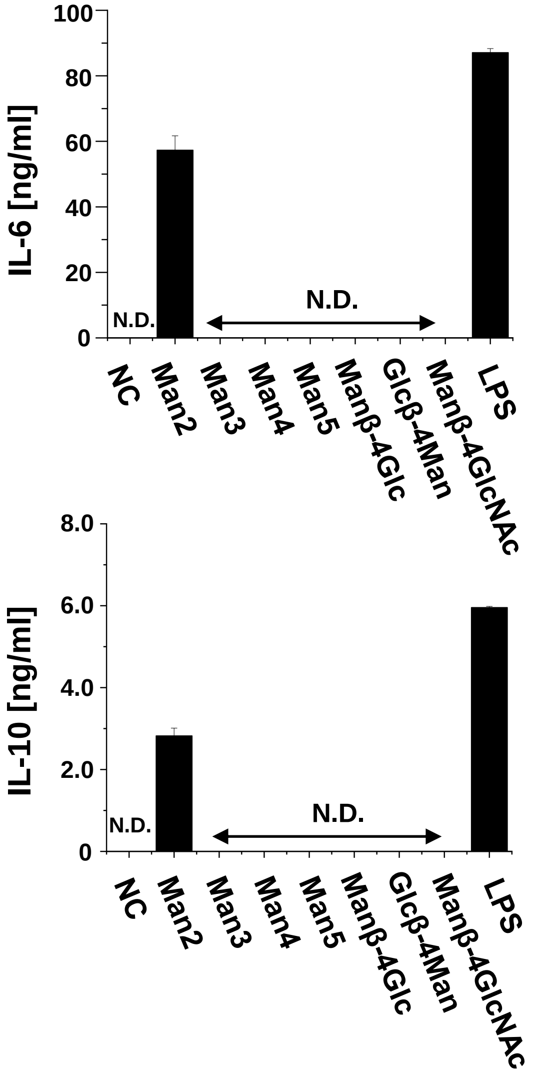

Fig. S4. Stimulatory effect of mannooligosaccharides on cytokine production in BMDCs. BMDCs ($1x10^6$ cells/mL) derived from BALB/c mice were stimulated with 50 μM of the indicated mannooligosaccharides or 1.0 μg/mL of LPS. The concentrations of IL-6 (upper graph) and IL-10 (lower graph) in the culture supernatants were measured by ELISA. The experiment was performed once. The similar trend was observed in BMDCs derived from C57BL/6 mice. N.D. (not detectable): <31.25 pg/mL

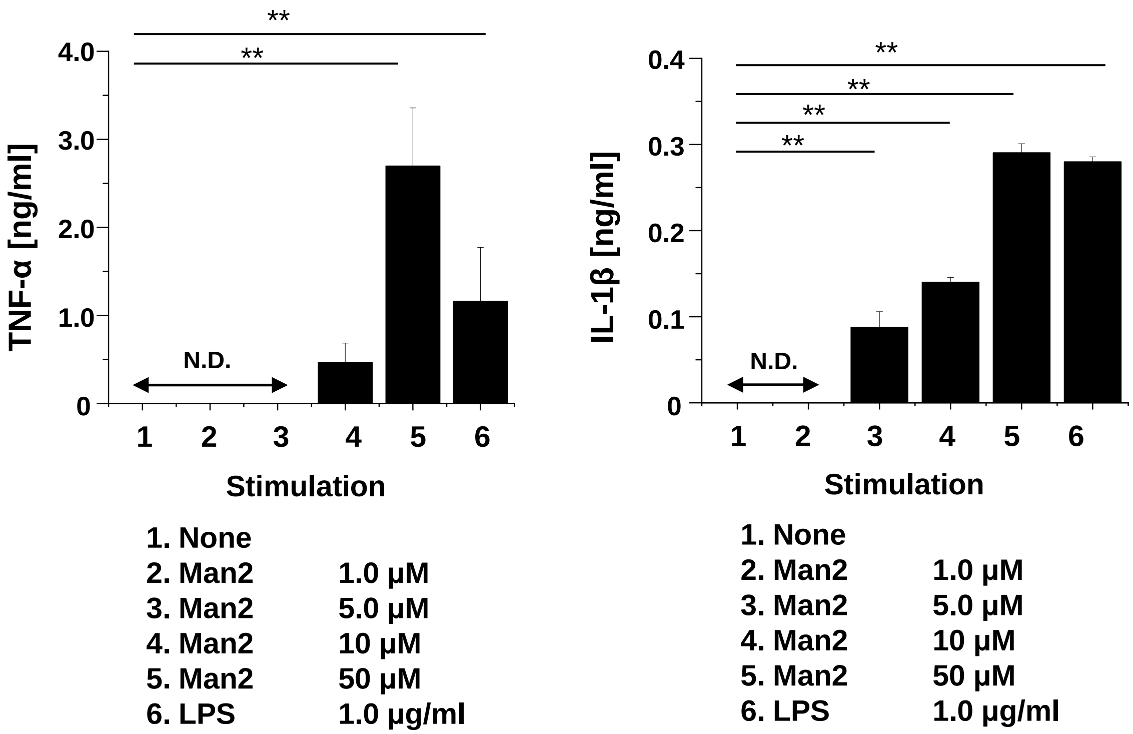

Fig. S5. Stimulatory effect of Man2 on TNF-α and IL-1β production in BMDCs. BMDCs derived from C57BL/6 mice ($1x10^6$ cells/mL) were stimulated with various concentrations of Man2 or 1.0 μg/mL of LPS. The concentrations of TNF-α (left graph) and IL-1β (right graph) in the cell culture supernatants were measured by ELISA. The data are representative for two independent experiments. **P<0.01 in Dunnett's test. All bar graphs show mean +/- SD. N.D. (not detectable): <31.25 pg/mL (TNF-α), <15.6 pg/ml (IL-1β)

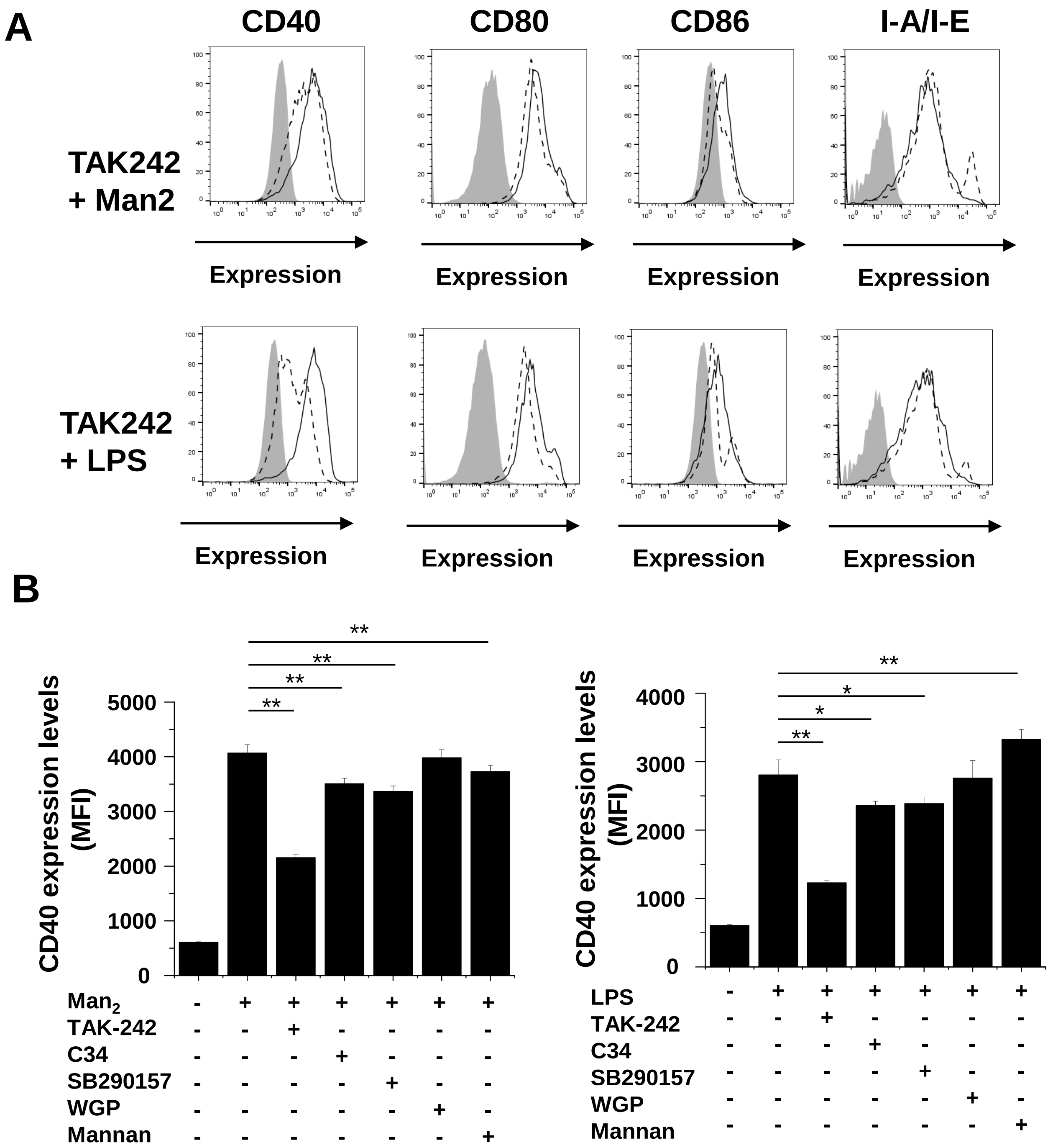

Fig. S6. Effect of receptor inhibitors on expressions of co-stimulatory molecules in Man2-treated BMDCs. BMDCs derived from C57BL/6 mice ($1x10^6$ cells/mL) were treated with either 100 nM TAK-242, 50 µM C34, 100 µM SB290157, 100 µg/mL of WGP® Soluble, or 200 µg/mL of mannan for 30 min and subsequently stimulated with 50 µM Man2 or 5.0 ng/mL of LPS for 24 hours. (A) Expression levels of CD40, CD80, CD86 or I-A/I-E on the cell surface of TAK-242-treated and Man2- or LPS-stimulated cells were analyzed by FACS. Grey area: unstimulated and unstained cells, dashed lines: inhibitor-and Man2- or LPS-treated and mAb-stained cells, solid lines: inhibitor-untreated, Man2- or LPS-treated and mAb-stained cells. (B) Mean fluorescence intensity (MFI) of CD40 expression in inhibitor-treated and Man2, or LPS-stimulated cells were estimated. The data are representative for two independent experiments. *P<0.05 , **P<0.01 in Dunnett's test. All bar graphs show mean +/- SD.

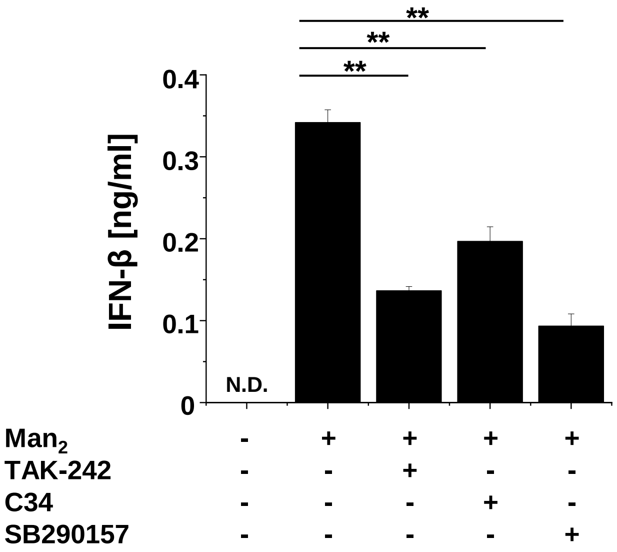

Fig. S7. Effect of receptor inhibitors on expressions of IFN-β production in Man2-treated BMDCs. BMDCs derived from C57BL/6 mice ($1x10^6$ cells/mL) were treated with 100 nM TAK-242, 50 μM C34, or 100 μM SB290157 for 30 min, and subsequently stimulated with 50 μM Man2 for 24 hours. The concentrations of IFN-β in the culture supernatants were measured by ELISA. The data are representative for two independent experiments. **P<0.01 in Dunnett's test. All bar graphs show mean +/- SD. N.D. (not detectable): <25.0 pg/mL

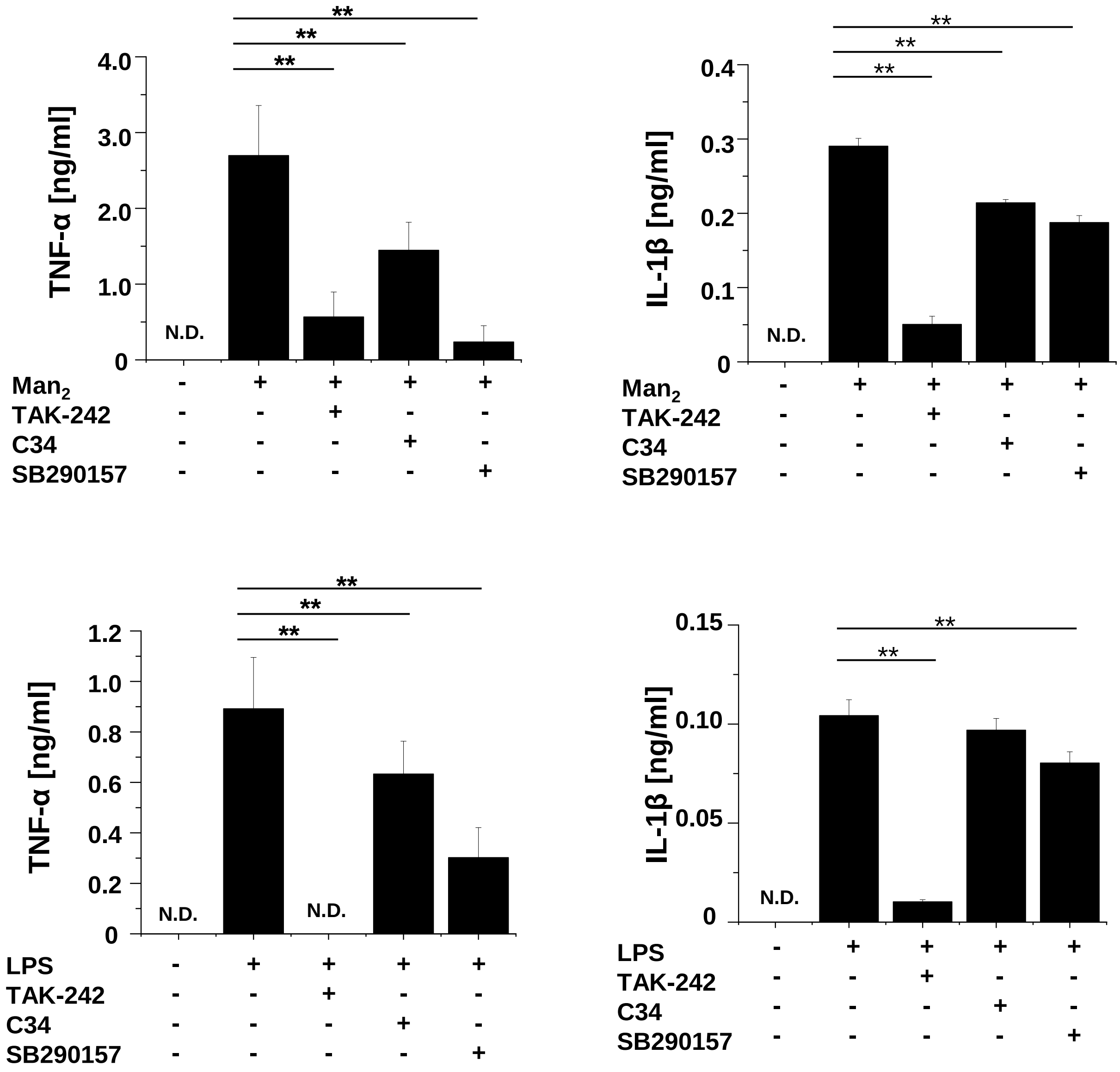

Fig. S8. Effect of receptor inhibitors on expressions of TNF-α and IL-1β production in Man2-treated BMDCs. BMDCs derived from C57BL/6 mice (1x10^6 cells/mL) were treated with 100 nM TAK-242, 50 μM C34, or 100 μM SB290157 for 30 min, and subsequently stimulated with 50 μM Man2 or 5.0 ng/mL of LPS for 24 hours. (A) The concentrations of TNF-α and (B) IL-1β in the culture supernatants were measured by ELISA. The data are representative for two independent experiments. All bar graphs show mean +/- SD. N.D. (not detectable): <31.25 pg/mL (TNF-α), <15.6 pg/mL (IL-1β)

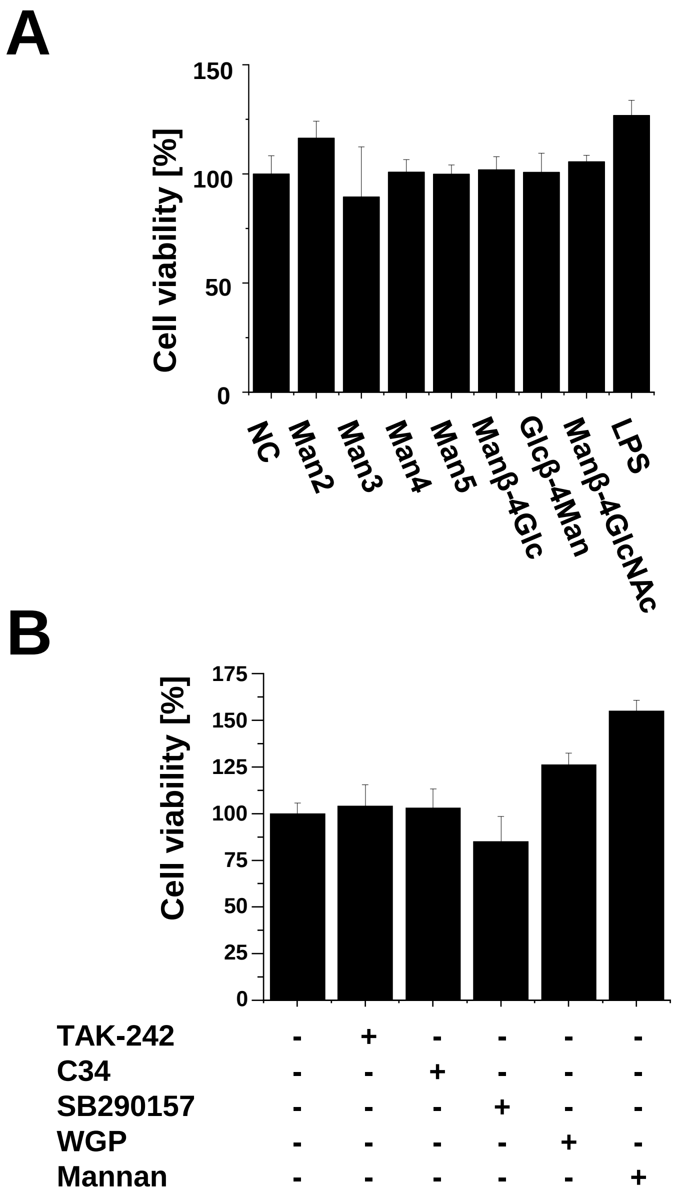

Fig. S9. The influence of receptor inhibitors on viability of BMDCs. (A) BMDCs derived from C57BL/6 mice (1x10^{6} cells/mL) were stimulated with 50 μM mannooligosaccharide or 1.0 μg/mL of LPS for 24 hours. (B) BMDCs derived from C57BL/6 mice (1x10^{6} cells/mL) were treated with 100 nM TAK-242, 50 μM C34, 100 μM SB290157, 100 μg/mL of WGP® Soluble, or 200 μg/mL of mannan for 30 min, and subsequently stimulated with 10 μM Man2 or 1.0 μg/mL of LPS for 24 hours. CCK-8 assay was performed to assess the influence of mannooligosaccharides and receptor inhibitors on the viability of BMDCs. The data are representative for two independent experiments. All bar graphs show mean +/- SD.
